# Construction of a Genetic Linkage Map and Genetic Analysis of Domestication Related Traits in Mungbean (*Vigna radiata*)

**DOI:** 10.1371/journal.pone.0041304

**Published:** 2012-08-02

**Authors:** Takehisa Isemura, Akito Kaga, Satoshi Tabata, Prakit Somta, Peerasak Srinives, Takehiko Shimizu, Uken Jo, Duncan A. Vaughan, Norihiko Tomooka

**Affiliations:** 1 Biodiversity Research Unit, Genetic Resources Center, National Institute of Agrobiological Science, Tsukuba, Ibaraki, Japan; 2 Department of Plant Genome Research, Kazusa DNA Research Institute, Kisarazu, Chiba, Japan; 3 Department of Agronomy, Faculty of Agriculture at Kamphaeng Saen, Kasetsart University, Nakhon Pathom, Thailand; 4 Research Division 1 for Plants, Institute of Society for Techno-Innovation of Agriculture, Forestry and Fisheries, Tsukuba, Ibaraki, Japan; Nanjing Forestry University, China

## Abstract

The genetic differences between mungbean and its presumed wild ancestor were analyzed for domestication related traits by QTL mapping. A genetic linkage map of mungbean was constructed using 430 SSR and EST-SSR markers from mungbean and its related species, and all these markers were mapped onto 11 linkage groups spanning a total of 727.6 cM. The present mungbean map is the first map where the number of linkage groups coincided with the haploid chromosome number of mungbean. In total 105 QTLs and genes for 38 domestication related traits were identified. Compared with the situation in other *Vigna* crops, many linkage groups have played an important role in the domestication of mungbean. In particular the QTLs with high contribution were distributed on seven out of 11 linkage groups. In addition, a large number of QTLs with small contribution were found. The accumulation of many mutations with large and/or small contribution has contributed to the differentiation between wild and cultivated mungbean. The useful QTLs for seed size, pod dehiscence and pod maturity that have not been found in other Asian *Vigna* species were identified in mungbean, and these QTLs may play the important role as new gene resources for other Asian *Vigna* species. The results provide the foundation that will be useful for improvement of mungbean and related legumes.

## Introduction

Mungbean [*Vigna radiata* (L.) Wilczek] is one of the most important grain legumes in Asian agriculture, particularly in South Asia [1]. Almost 90% of world’s mungbean production comes from Asia, and India is the world’s largest producer [2]. Mungbean is grown on about 4.2 million hectares in India with an annual average production of 1.3 million tons in 2008 [3]. In Pakistan, mungbean is grown on 250 thousand hectares with an average annual production of 180 thousand tons in 2009 [4]. In Thailand, although the area planted to mungbean has decreased over the past 15 to 20 years, mungbean is currently sown on 200 thousand hectares and it is the most economically and socially important legume [5]. Mungbean in China is grown on about 1 million hectares and its production and export amounts have increased in recent years [6].

In South Asian countries, mungbean is an important protein source that usually consumed as a soup of split seeds and spices called Dal or Dhal. In the countries of Southeast and East Asia, mungbean sprouts are consumed as a vegetable rich in vitamins and minerals [1]. Due to the importance of mungbean in Asian countries, the genetic improvement of mungbean is being carried out at the Word Vegetable Center (formerly the Asian Vegetable Research and Development Center, AVRDC). To date, over 110 mungbean cultivars have been released by AVRDC in South and Southeast Asia and around the world [7].

Genetic linkage maps of some mungbean have been constructed using RFLP, RAPD and SSR markers [8]–[15]. These linkage maps have been used to map genes for azuki bean weevil resistance [16]–[18] and seed color [19] and to identify QTLs for seed weight [8], [14], [18], hard-seededness [14], powdery mildew resistance [13], [20]–[22] and Cercospora leaf spot resistance [15].

Despite many genetic linkage maps have been developed, the numbers of linkage groups reported have not coincided with the basic number of chromosomes in mungbean (*n*  = 11). In addition, the populations that were used to construct previous maps were small, and there were large distances between adjacent markers in some linkage groups.

In addition to mungbean, the *Vigna* species that were domesticated in Asia include three other important legume crops: azuki bean [*Vigna angularis* (Willd.) Ohwi and Ohashi], black gram [*V. mungo* (L.) Hepper], and rice bean [*V. umbellata* (Thunb.) Ohwi and Ohashi] [1]. Numerous differences in morphological and physiological traits associated with domestication are observed between cultivated and wild forms in these species. These four species are closely related members of the same subgenus, *Ceratotropis* [1], and they provide ideal materials for comparative analyses of domestication genomics. The information from these analyses will elucidate the genetics of domestication within the subgenus *Ceratotropis* and enable the isolation of novel genes for use in breeding. For these purposes, we have previously published results of QTL analyses for azuki bean [23], [24] and rice bean [25].

In this paper, we focus on the domestication of mungbean as a part of our comparative genome analysis among domesticated species in the subgenus *Ceratotropis*. The presumed wild ancestor of mungbean is *Vigna radiata* var. *sublobata* (Roxb.) Vercourt, which is distributed across the Old World tropics from western Africa to northern Australia and Papua New Guinea [1]. Mungbean has a long cultivation history and considerable diversity in South Asia [26], and it is believed that mungbean was domesticated in South Asia [1], [27]–[28].

The objectives in this study were (1) to construct a genetic linkage map using SSR markers from azuki bean, mungbean, cowpea, and common bean (*Phaseolus vulgaris*) and EST-SSR markers from soybean (*Glycine max*), using a BC_1_F_1_ population derived from a cross between cultivated and wild mungbean; (2) to identify the genome regions associated with domestication traits in mungbean; and (3) to compare the results with those previously obtained for azuki bean and rice bean.

## Materials and Methods

### Mapping Population

A BC_1_F_1_ mapping population consisting of 250 individuals was developed from a cross between a wild mungbean accession (JP211874) collected in Myanmar and a cultivated mungbean accession (JP229096 cv. Sukhothai), a landrace collected from Thailand. The cultivated accession was used as the recurrent parent and as the male parent in the crosses to obtain the F_1_ and BC_1_F_1_ plants. The wild accession was obtained from the National Institute of Agrobiological Sciences (NIAS) Gene Bank, Tsukuba, Japan, and the cultivated accession was obtained from Kasetsart University, Thailand.

### DNA Extraction

Total genomic DNA of each BC_1_F_1_ plant was extracted from 200 mg of fresh leaf tissue using a DNeasy Plant Mini Kit (QIAGEN, Valencia, CA, USA). DNA concentration was adjusted to 5 ng/µl for SSR and EST-SSR analyses by comparing each sample with known concentrations of standard λ DNA in 1% agarose gel.

### SSR Screening and Analysis

In this study, 329 SSR primer pairs for azuki bean [29]; 50 from mungbean, including two markers associated with bruchid beetle resistance [30]–[33]; seven from cowpea [34]; and 40 from common bean [35]–[38] were screened for polymorphism between the two parents. By using the read2Marker program [39], we designed another 156 cowpea SSR primer pairs from cowpea genomic sequences in the Cowpea Genomics Knowledge Base (CGKB) database ([40]; http://cowpeagenomics.med.virginia.edu/CGKB/) and screened them for polymorphism between the mapping parents. The information on the primers that are mapped in this study is shown (Table S1). The SSR analysis was carried out according to the method of Han et al. [41].

In this study, 840 EST-SSR primer pairs from soybean [42] were screened for polymorphism between the two parents (Table S1.). The 5′-end of each forward primer was labeled with one of three fluorescent dyes: FAM, HEX or NED (Applied Biosystems, Foster City, CA, USA). To amplify the fragments from multiple primer pairs in a PCR reaction, a QIAGEN Multiplex PCR Kit (QIAGEN) was used. Five microliters of PCR reaction mixture contained 5 ng genomic DNA, 1× QIAGEN Multiplex PCR Master Mix, 1× Q-solution, 2 pmol of each forward primer and 20 pmol of each reverse primer. PCR amplifications were performed by the modified ‘Touchdown PCR’ program described by Sato et al. [43] using the following reaction conditions: 15 min at 95°C for initial denaturation, 3 cycles of 30 sec at 94°C and 3 min at 59°C, followed by 3 rounds of the same program in which the annealing temperatures were decreased by 3°C every 3 cycles, then 2 rounds of a 3-step program of 3 cycles of 30 sec at 94°C, 3 min at 48°C, 1 min at 72°C, followed by 40 cycles of 30 sec at 94°C, 3 min at 45°C, 1 min at 72°C, with a final extension for 10 min at 72°C. One microliter of PCR product was mixed with 13 µl of Hi-Di formamide containing 0.25 µl Gene Scan 500 ROX size standard (Applied Biosystems) and run on an ABI Prism 3100 Genetic Analyzer (Applied Biosystems). Alleles were determined according to the SSR analysis method of Han et al. [41].

### Map Construction

The linkage map was constructed according to the method of Han et al. [41]. JoinMap ver. 4.0 [44] was used to construct the linkage map. The segregation of each marker was analyzed by a chi-square test for goodness of fit to the expected Mendelian ratio for a backcross (1∶1). The recombination frequencies were converted into map distances (cM) using the Kosambi mapping function [45]. The numbering of linkage groups follows that for azuki bean [41].

### Trait Measurement

A total of 38 traits related to domestication were evaluated (Table 1). Of these, 34 were treated as quantitative traits and four [seed coat color (SDC), black mottle on seed coat (SDCBM), hilum color (SDHC), and stem determinacy (STDET)] were treated as qualitative traits. The BC_1_F_1_ population of 250 plants, together with 12 plants of each parent, was grown in the vinyl house at NIAS, Tsukuba, Japan, 36°2′N, 140°8′E, from July to December 2007 under natural day length. The natural day length gradually decreased from 14 h 12 min (24^th^ July) to 9 h 43 min (20^th^ December). The temperature in the vinyl house during growth period ranged from 20°C to 35°C. From October, the vinyl house was heated to maintain a minimum temperature of 20°C.

**Table 1:** Domestication-related traits examined in the BC1F1 (or BC1F1∶2) populations derived from a cross between cultivated mungbean and its presumed wild ancestor.

General attribute	Organ	Trait	Trait abbreviation	QTL/gene	Evaluationmethod	Evaluated population
Seed dormancy	Seed	Water absorption (%)	SDWA	*Sdwa*	Percent of seeds that absorbedwater at five day after sowingat 15°C in incubator	BC_1_F_1∶2_
Pod dehiscence	Pod	Number of twist (count)	PDT	*Pdt*	Number of twists along the lengthof the shattered pod	BC_1_F_1_
		Rate of shattered pods (%)	PDR1W, 2 W, 4 W	*Pdr1 w, 2 w, 4 w*	Percent of shattered pods to totalpods at one, two and four week(s)after harvesting	BC_1_F_1_
Gigantism	Seed	100 seed-weight (g)	SD100WT	*Sd100wt*	Weight of 100 seeds	BC_1_F_1∶2_
		Length (mm)	SDL	*Sdl*	Maximum distance from top tobottom of the seed	BC_1_F_1∶2_
		Width (mm)	SDW	*Sdw*	Maximum distance from hilum toits opposite side	BC_1_F_1∶2_
		Thickness (mm)	SDT	*Sdt*	Maximum distance between bothsides of the hilum	BC_1_F_1∶2_
	Pod	Length (cm)	PDL	*Pdl*	Length of straight pod	BC_1_F_1_
		Width (mm)	PDW	*Pdw*	Maximum width	BC_1_F_1_
	Stem	Thickness (mm)	STT	*Stt*	Stem diameter under the primaryleaf	BC_1_F_1_
	Leaf	Primary leaf length (cm)	LFPL	*Lfpl*	Distance from pulvinus to leaf tip	BC_1_F_1_
		Primary leaf width (cm)	LFPW	*Lfpw*	Maximum width	BC_1_F_1_
		Maximum leaflet length (cm)	LFML	*Lfml*	Length of the largest terminalleaflet on leaves between node onfirst trifoliate leaf and node ontenth trifoliate leaf	BC_1_F_1_
		Maximum leaflet width (cm)	LFMW	*Lfmw*	Width of the largest terminalleaflet on leaves between node onfirst trifoliate leaf and node ontenth trifoliate leaf	BC_1_F_1_
Plant type	Hypocotyl Epicotyl	Hypocotyl plus epicotyl length (cm)	HECL	*Hecl*	Length from surface of soil toprimary leaf	BC_1_F_1_
	Stem	Internode length (first to tenth) (cm)	ST1I-ST10I	*St1i -St10i*	Length from node on primaryleaf to each node	BC_1_F_1_
		Length (cm)	STL	*Stl*	Length from node on primary leafto node on tenth trifoliate leaf	BC_1_F_1_
		Determinacy	STDET	*stdet*	Indeterminate (wild) or determinate(cultivated)	BC_1_F_1_
	Branch	Number (count)	BRN	*Brn*	Number of branches on main stem	BC_1_F_1_
		Position of first branch (*i*th node)	BRP	*Brp*	Position of first branch on mainstem	BC_1_F_1_
Phenology	Flower	Days to first flower (day)	FLD	*Fld*	Number of days from sowing to flowering of first flower	BC_1_F_1_
	Pod	Days to maturity of first pod (day)	PDDM	*Pddm*	Number of days from flowering offirst flower to harvest of first pods	BC_1_F_1_
Yield potential	Seed	Number of seed per pod (seed/pod)	SDNPPD	*Sdnppd*	Number of seed per pod	BC_1_F_1∶2_
	Pod	Total number (pod)	PDTN	*Pdtn*	Total number of pod harvested	BC_1_F_1_
Pigmentation	Seed	Seed-coat color	SDC	*sdc*	Brown (wild) or Green (cultivated)	BC_1_F_1∶2_
		Black mottle on seed-coat	SDCBM	*sdcbm*	Present (wild) or absent (cultivated)	BC_1_F_1∶2_
		Hilum color	SDHC	*sdhc*	Pale red (wild) or White (cultivated)	BC_1_F_1∶2_

The seedling traits [primary leaf length (LFPL), primary leaf width [LFPW], hypocotyl plus epicotyl length (HECL)] were recorded when the 1st trifoliate leaf opened, and the vegetative traits [maximum leaf length (LFML), maximum leaf width (LFMW), lengths of the 1st through 10th internodes (ST1I–ST10I), stem length (STL), and stem thickness (STT)] were recorded when the 10th trifoliate leaf was fully developed. After the final flowers bloomed, the determinacy (STDET) was recorded. After all pods were harvested, branch number (BRN), position of first branch on main stem (BRP), and total pod number (PDTN) were recorded. These traits were evaluated in the BC_1_F_1_ generation.

The seed-related traits were investigated using the seeds from BC_1_F_1_ plants. *i.e.*, the BC_1_F_1∶2_ generation. The water absorption by seed (SDWA) was measured as an index of seed dormancy. Fifteen unscarified seeds were placed on wet filter paper and incubated in the dark at 15°C for 5 d, and the number of seeds that imbibed water was counted. Seed length, weight, and thickness (SDL, SDW and SDT, respectively) were the average of 10 seeds. The 100-seed weight (SD100WT) was evaluated using only intact seeds. The number of seeds per pod (SDNPPD) was measured using 10 pods.

The pod traits, namely pod length (PDL), pod width (PDW) and number of twists along the length of the dehisced pod when kept at room temperature (PDT), were measured on 10 pods. PDT was used as one index of pod dehiscence. As a second index of pod dehiscence, the percent of shattered pods out of total pods at 1, 2 and 4 weeks after harvesting was recorded (PDR1 W, −2 W and −4 W, respectively).

The number of days from sowing to first flowering (FLD) and the days from first flowering to harvesting of the first pod (PDDM) were recorded in the BC_1_F_1_ population.

The seed coat color (SDC), black mottle on seed coat (SDCBM) and hilum color (SDHC) were evaluated using seeds from the BC_1_F_1_ plants (BC_1_F_1∶2_). These seed color traits are maternally inherited, so the seed phenotypes reflect the genetic constitution of the parent plant.

### Data Analysis

For the quantitative traits, the mean, standard error and broad-sense heritability were calculated, and the frequency distribution of phenotypes in the BC_1_F_1_ (or BC_1_F_1∶2_) population was examined for each trait. The correlation coefficient between each pair of traits was also calculated. For the four qualitative traits, the phenotypes were investigated in each BC_1_F_1_ or BC_1_F_1∶2_ individuals and the frequency distribution was analyzed by a chi-square test for goodness of fit to the expected Mendelian ratio (1∶1). The segregation pattern data were used to identify the map position of the gene controlling each qualitative trait.

### QTL Analysis

Untransformed data for each quantitative trait except for SDWA, PDR1W, PDR2W, and PDR4W were used in QTL analysis. The data for these four traits were arcsine-transformed before use in the QTL analysis. QTLs were identified as described by Kaga et al. [24] using the software package MultiQTL ver. 2.5 (http://www.multiqtl.com/). The QTL nomenclature followed the style of Isemura *et al.* [23], [25] and Kaga et al. [24]. The trait abbreviation (Table 1) is followed by the population number (5), linkage group and then QTL number (1 or 2). For the population number, No.1– No.3 were used in azuki bean populations [23], [24] and No.4 was used in rice bean population [25]. For QTL number, when two QTLs for a trait were detected on same one linkage group, these QTLs were distinguished as No.1 and 2.

A chi-square goodness-of-fit test for observed number of domestication related QTL to the expected number of QTL across each linkage group was applied to determine the random distribution of QTL with the assumption of independent gene action. To test whether or not QTL were randomly distributed along a linkage group, a Poisson distribution function *P(x)  =  e^-^* ^μ^µ^x^ */x!*, where x is the number of QTL per 10-cM interval and μ is the average QTL density on linkage groups, was calculated.

## Results

### Analysis with SSR and EST-SSR Markers

SSR primers developed from azuki bean, mungbean, cowpea and common bean and EST-SSR primers from soybean were used to construct a genetic linkage map of mungbean. The amplification ratio of SSR and EST-SSR primers from the five legumes was comparatively high in mungbean, with values between 76.7% (cowpea primers) and 98.0% (mungbean primers) (Table 2). Out of the 1422 SSR and EST-SSR primer pairs screened, 128 azuki bean, 27 mungbean, 56 cowpea, nine common bean and 155 soybean primer pairs revealed clear polymorphism between the parents of the mapping population (Table 2). The percentage of primer pairs that revealed polymorphism between the parents was highest in mungbean (54.0%); the percentage decreased in relation to the phylogenetic distance of the species to mungbean and was lowest in soybean (18.5%) (Table 2). All 430 marker loci detected by the 375 primer pairs were used for the construction of the mungbean map (The genotype data of 430 marker loci is shown in Dataset S1).

**Table 2:** Summary of amplification and polymorphic rate in mungbean of SSR and EST-SSR primer pairs from five legumes.

	No. of SSR and EST-SSR primer pairs			No. of mapped loci
	Screened	Amplified (%)	Polymorphic (%)	
mungbean	50	49 (98.0)	27 (54.0)	30
azuki bean	329	258 (78.4)	128 (38.9)	140
cowpea	163	125 (76.7)	56 (28.6)	58
common bean	40	34 (85.0)	9 (22.5)	9
soybean	840	668 (79.5)	155 (18.5)	193
Total	1422	1134 (79.7)	375 (26.4)	430

### Linkage Map Construction

**Figure 1:**
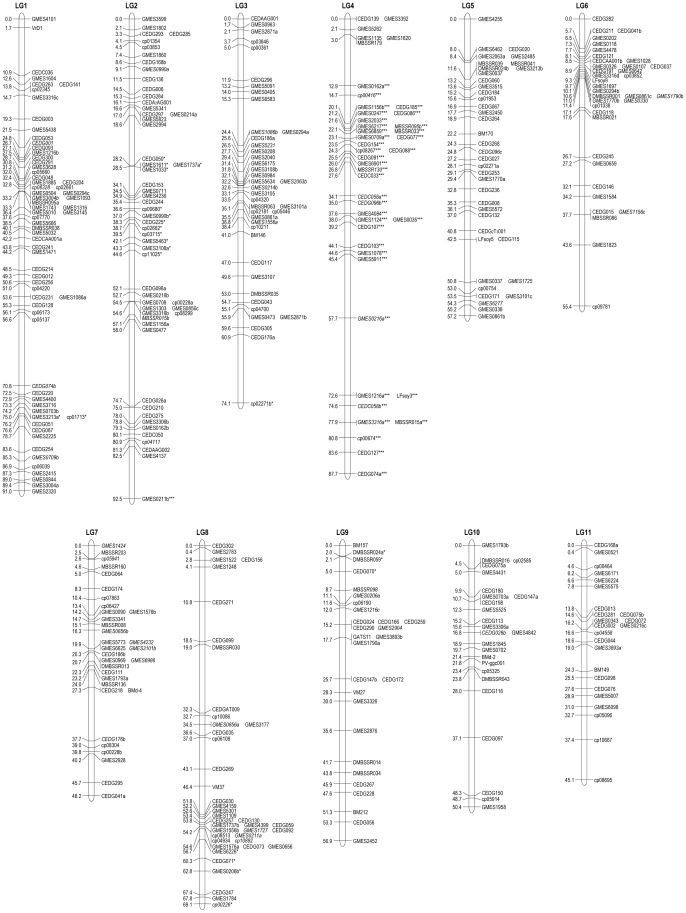
Genetic linkage map of mungbean based on SSR markers from mungbean and related species. Map distances and marker names are shown on the left and right side of the linkage groups, respectively. Lower-case letters following marker names indicate multiple loci detected by the same primer pairs. Marker names in italics indicate dominant loci. Markers showing significant deviation from the expected segregation ratio at the 0.05, 0.01, and 0.001 levels are indicated with *, **, and ***, respectively. SSR markers with prefixes CED are derived from azuki bean. Markers with prefixes DMBSSR, MBSSR and VrD1 are derived from mungbean. Markers with prefixes cp and VM are derived from cowpea. Markers with prefixes BM, BMd-, GATS11 and PV-ggc001 are derived from common bean. Markers with prefixes GMES and LFsoy are derived from soybean.

A total of 430 loci (140 azuki bean SSR loci, 30 mungbean SSR loci, 58 cowpea SSR loci, nine common bean SSR loci and 193 soybean EST-SSR loci) could be assigned to 11 linkage groups (LGs) covering a total of 727.6 cM of the mungbean genome at an average marker density of 1.78 cM (Figure 1 and Table 3). The genome region represented by this map covered 87.4% of the azuki bean genome (832.1 cM) reported by Han et al. [41]. The number of markers on each linkage group ranged from 24 (LG11) to 65 (LG1). The length of each linkage group ranged from 45.1 cM (LG11) to 92.5 cM (LG2). The average distance between adjacent markers ranged from 1.42 cM (LG1) to 2.11 cM (LG9). At least one gap of more than 10 cM between adjacent markers was present on each linkage group except for LG5, LG9 and LG11, and LG2 had one gap greater than 15 cM. Segregation distortion (*P*<0.05) was observed for 61 markers (14.4%). The frequency of plants with a heterozygous genotype was unexpectedly high for 58 of the 61 markers showing segregation distortion, and most of these were found on LG2 and LG4. Almost all markers on LG4 showed a high level of segregation distortion (*P*<0.001).

**Table 3:** Number of markers and average distance between markers on each linkage group in the mungbean linkage map.

Linkage Group (LG)	Length (cM)	No. of markers						The distance between two maker loci (cM)
		azuki bean	mungbean	cowpea	common bean	soybean	Total	
		SSR	SSR	SSR	SSR	EST-SSR		
1	91.0	23	3	10	0	29	65	1.42
2	92.5	18	1	9	0	27	55	1.71
3	74.1	7	2	8	1	23	41	1.85
4	87.7	15	5	3	0	21	44	2.04
5	57.2	15	3	3	1	17	39	1.51
6	55.4	11	3	3	0	19	36	1.59
7	48.2	8	5	5	1	13	32	1.55
8	69.1	15	1	7	0	18	41	1.73
9	56.9	10	5	2	3	8	28	2.11
10	50.4	9	2	3	2	9	25	2.10
11	45.1	9	0	5	1	9	24	1.96
Total	727.6	140	30	58	9	193	430	1.78

### Field Data Analysis and Correlations between Traits

The means, standard errors, and broad-sense heritability of traits in the parental lines and BC_1_F_1_ plants or BC_1_F_1∶2_ lines are shown in Table 4 (The phenotype data of BC_1_F_1_ plants or BC_1_F_1∶2_ lines for each trait are shown in Dataset S2).

**Table 4:** Mean, standard error and heritability values for parents and the BC_1_F_1_ (or BC_1_F_1∶2_) populations derived from a cross between cultivated mungbean and its presumed wild ancestor.

	P1 (wild)		P2 (cultivated)		BC_1_F_1_ or BC_1_F_1∶2_ b		Heritability	
Trait^a^	Mean	SE^c^	Mean	SE^c^	Mean	SE^c^	(%)	
SD100WT (g)	1.17	0.03	6.89	0.12	4.81	0.05	86.3	
SDL (mm)	2.84	0.01	5.36	0.02	4.64	0.02	98.5	
SDW (mm)	2.22	0.01	4.12	0.01	3.74	0.02	99.2	
SDT (mm)	2.27	0.01	4.12	0.01	3.76	0.02	98.5	
SDWA (%)	1.3	1.33	100.0	0.00	38.7	2.0	99.6	
SDNPPD (seed/pod)	9.24	0.13	13.53	0.19	9.99	0.09	85.2	
SDCBM	Present		Absent		P:A = 146∶104 (x^2^ = 7.056*)			
SDC	Brown		Green		B:G = 122∶128 (x^2^ = 0.144)			
SDHC	Pale red		White		P:W = 124∶126 (x^2^ = 0.016)			
PDR1W (%)	83.6	2.74	0.0	0.00	15.7	0.91	79.8	
PDR2W (%)	99.3	0.53	0.0	0.00	19.6	1.13	99.5	
PDR4W (%)	100.0	0.00	0.0	0.00	29.7	1.61	100.0	
PDT (count)	1.71	0.02	1.46	0.04	1.57	0.03	92.1	
PDL (cm)	3.97	0.02	11.88	0.08	8.44	0.07	96.8	
PDW (mm)	5.23	0.01	8.74	0.02	8.18	0.04	98.9	
PDTN (pod)	39.0	4.93	30.8	3.70	57.3	1.58	77.1	
LFPL (cm)	3.45	0.04	9.31	0.07	6.59	0.04	91.5	
LFPW (cm)	2.08	0.02	3.14	0.04	2.66	0.02	84.9	
LFML (cm)	8.36	0.17	16.88	0.61	13.53	0.13	43.0	
LFMW (cm)	7.55	0.13	13.46	0.62	11.78	0.13	40.2	
STT (mm)	4.82	0.08	8.75	0.18	7.60	0.06	70.2	
HECL (cm)	2.1	0.05	7.8	0.17	4.7	0.07	83.7	
ST1I (cm)	0.2	0.02	1.8	0.07	0.9	0.02	45.6	
ST2I (cm)	0.4	0.03	2.3	0.12	1.2	0.02	32.6	
ST3I (cm)	0.6	0.03	3.0	0.13	1.8	0.04	65.0	
ST4I (cm)	1.2	0.03	5.3	0.20	3.0	0.05	58.2	
ST5I (cm)	1.9	0.07	7.4	0.21	4.7	0.07	79.3	
ST6I (cm)	2.7	0.11	10.7	0.32	6.0	0.10	71.0	
ST7I (cm)	6.2	0.33	12.8	0.39	7.4	0.12	55.0	
ST8I (cm)	7.0	0.34	16.1	0.27	9.7	0.19	88.1	
ST9I (cm)	31.2	0.70	13.3	0.44	14.3	0.34	86.5	
ST10I (cm)	39.1	0.99	10.2	0.46	19.6	0.52	90.4	
STL (cm)	92.6	1.03	90.6	1.51	73.2	1.14	94.0	
STDET	Indeterminate		Determinate		I:D = 135∶115 (x^2^ = 1.600)			
BRN (count)	6.0	0.23	2.7	0.23	5.2	0.16	91.0	
BRP (*i*th node)	2.0	0.00	4.0	0.30	3.5	0.13	85.9	
FLD (day)	80.9	0.93	35.4	0.34	56.2	0.59	93.9	
PDDM (day)	33.0	0.76	20.9	0.26	29.5	0.58	95.7	

a Trait abbreviation are shown in Table 1.

b Evaluated population for each trait are shown in Table 1.

c SE: Standard error.

* indicate significance at 5% level.

The BC_1_F_1_ plants and BC_1_F_1∶2_ lines showed a high degree of morphological and physiological variation (Table 4). High heritability (over 80%) was observed for most seed- and pod-related traits, whereas low heritability (below 70%) was observed for many stem- and leaf-related traits. The means of the BC_1_F_1_ plants and BC_1_F_1∶2_ lines fell between the means of cultivated and wild parents for all traits except for stem length (STL) and total pod number (PDTN). Most traits showed a nearly normal distribution among lines (or plants) in these populations (Figure S1 and S2). Transgressive segregation was observed for SDNPPD, PDT, PDTN, ST7I, ST8I, ST9I, STL, BRN, BNP LFMW and PDDM. Out of the four qualitative traits, the segregation ratios for seed coat color (SDC), hilum color (SDHC) and determinacy (STDET) fit the expected ratio (1∶1). For black mottle on seed coat (SDCBM), the segregation ratio was distorted from the expected ratio (χ^2^ = 7.056, *P*<0.05) because the frequency of the wild-parent phenotype was higher than expected.

In general, there were significant positive correlations (*P*<0.05) between similar or related traits such as between stem length and each internode length and between seed size–related traits (Table S2). In most cases, seed size–related traits (SD100WT, SDL, SDW and SDT) were positively correlated with seed coat permeability (SDWA), pod size–related traits (PDL and PDW), hypocotyl plus epicotyl length (HECL) and internode length (ST1I–ST8I). Seed size related-traits were negatively correlated with pod dehiscence–related traits (PDR1W, PDR2W, PDR4W and PDT), days to first flowering (FLD) and days to harvesting of first pod from first flowering (PDDM). SDWA was negatively correlated with pod dehiscence traits (PDT, PDR1W, PDR2W and PDR4W). FLD was negatively correlated with upper internode length (ST8I, ST9I and ST10I) and stem length (STL), and positively correlated with PDDM and branch number (BRN).

### QTLs for Each Trait

**Figure 2:**
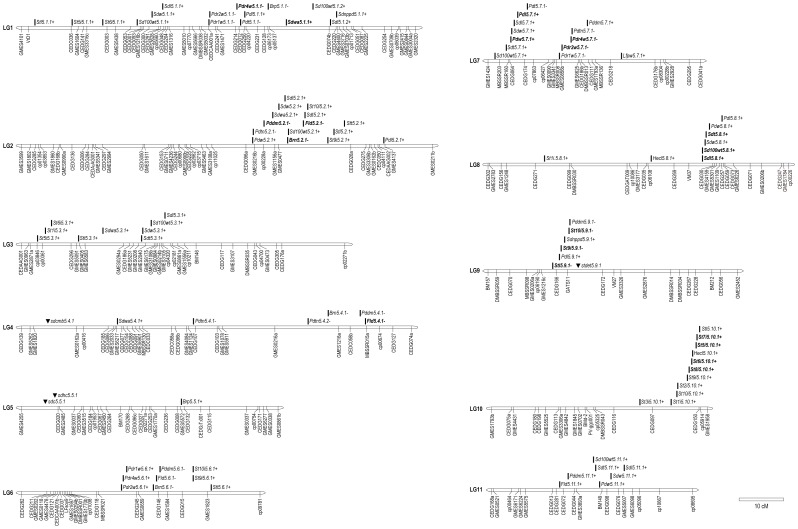
Distribution of the domestication-related QTLs and genes detected on each linkage group. The signs “▪”and “▾” indicate the position of detected QTLs and genes, respectively. The signs “+” and “-” after QTL and gene names indicate the direction of effect of alleles from cultivated mungbean. The QTLs in bold italic have the large effects (PVE ≥20%).

The results of QTL analyses for each trait in the population are shown (Figure 2 and Table S3). In total, 101 QTLs and four morphological marker genes were identified for 38 domestication-related traits. This number of QTLs overestimates the total number of QTLs due to the measurement of correlated traits. One to seven QTLs were detected for each trait at the level of significance (*P*<0.002) except for primary leaf length (LFPL), maximum leaf length (LFML), maximum leaf width (LFMW) and 4th internode length (ST4I), for which no QTLs were detected.

Seed dormancy (SDWA): A critical trait associated with domestication is the loss of seed dormancy. The percentage of the seeds that imbibed water by the assay for water absorption by seeds (SDWA) was used as an index of seed dormancy. The percentage of the seeds that imbibed water in the cultivated parent was higher than in the wild parent. It was expected that for QTLs detected for this trait, the alleles from the cultivated parent would increase seed coat permeability. Four QTLs were detected on LG1, LG2, LG3 and LG4. As expected, the alleles of the cultivated parent increased SDWA at all QTLs. The QTL on LG1 explained 33.7% of the phenotypic variation.

Pod dehiscence (PDT, PDR1W, PDR2W, PDR4W): Pods dehiscence reduces the number of seeds harvested. The number of twists along the length of the shattered pod (PDT) and the percentage of shattered pods one week (PDR1W), two weeks (PDR2W) and four weeks (PDR4W) after harvesting were used as indices of pod dehiscence. Two QTLs for PDT were found on LG1 and LG7, but the PDT values for the cultivated and wild parent were almost the same, so the contributions of these QTLs to this trait were low (12.4% and 10.8% on LG1 and LG7, respectively). As expected, the alleles from the cultivated parent decreased PDT. For PDR1W, PDR2W and PDR4W, there were considerable differences in the percentage of shattered pods between the parents. For each of these traits, three QTLs were detected at similar locations on LG1, LG6 and LG7. The alleles from the cultivated parent decreased the percentage of shattered pods at the QTLs on LG1 and LG7, whereas the allele from the cultivated parent increased the percentage of shattered pods at the QTLs on LG6. The phenotypic variation explained (PVE) values of QTLs on LG1 and LG7 increased as the length of time after harvesting increased. The QTLs for PDR4W on LG1 and LG7 explained over 20% of the phenotypic variation. The QTL positions for the three PDR traits on LG1 were the same as that for PDT, but the QTL positions for the three PDR traits on LG7 were about 10 cM away from the QTL position for PDT.

Seed size (SD100WT, SDL, SDW, SDT): Domestication of mungbean has resulted in about a six-fold increase in seed weight (Table 4). Five to seven QTLs were detected for seed size–related traits (SD100WT, SDL, SDW and SDT) on six linkage groups. The QTLs with the highest contributions to these traits (PVE  = 15.1%–22.7%) were found on LG8. The QTLs for the other seed size traits on LG2 also explained 11.4%–16.6% of the phenotypic variation. As expected, all QTLs alleles from the cultivated parent had a positive substitution effect.

Pod size (PDL, PDW): Domestication of mungbean has resulted in increased pod length and width (Table 4). Five QTLs for pod length (PDL) and four QTLs for pod width (PDW) were found, with QTLs affecting both traits on LG2, LG7 and LG8. The QTLs with the highest contribution to these traits [20.5% (PDL) and 28.5% (PDW)] were found at the same position on LG7. As expected, the alleles from the cultivated parent had the effect of increasing both PDL and PDW for all QTLs. The QTLs for both PDL and PDW on LG2, LG7 and LG8 were located close to QTLs for seed-related traits on these same linkage groups.

Leaf size (LFPL, LFPW, LFML, LFMW): For primary leaf length (LFPL), there was little variation in the BC_1_F_1_ plants based on the frequency distribution. As a result, no QTLs for this trait were detected. For primary leaf width (LFPW), only one QTL with a minor effect was detected on LG7 (PVE  = 7.5%). For maximum leaf length (LFML) and width (LFMW), the heritability values were low and no QTLs for these traits were detected.

Hypocotyl plus epicotyl length (HECL), internode length (ST1I–ST10I), and stem length (STL): The hypocotyl plus epicotyl length (HECL) and 1^st^ to 8^th^ internode lengths (ST1I–ST8I) of the cultivated parent were longer than those of the wild parent, whereas the 9^th^ and 10^th^ internode lengths (ST9I and ST10I) and stem length (STL) of the wild parent were longer than those of the cultivated parent (Table 4). The QTLs for HECL and ST1I–ST8I (except for ST4I, for which no QTLs were detected) were found on one or more of linkage groups 1, 3, 8 and 10. The QTLs for HECL and ST1I- ST10I (except for ST4I) were detected on LG10 and the QTLs for ST5I–ST8I on LG10 explained over 20% of the phenotypic variation (Figure 2 and Table S3). At all QTLs for HECL and ST1I–ST8I, alleles from the cultivated parent had the effect of increasing length. The QTLs for both ST9I and ST10I were found on LG2, LG6, LG9 and LG10; the QTLs on LG9 had the largest effects (PVE  = 22.5% and 47.7% for ST9I and ST10I, respectively). The QTLs for ST9I and ST10I on LG9 were located near the gene for determinate growth habit (Figure 2). The alleles from the cultivated parent had the effect of decreasing the lengths of ST9I and ST10I at the QTLs on LG9, whereas the alleles from the cultivated parent had the effect of increasing the lengths of these internodes at the QTLs on LG2, LG6 and LG10. The QTLs for STL were found on LG1, LG2, LG3, LG6, LG9 and LG10 at similar locations to the QTLs for internode length. The QTLs for STL on LG9 and LG10 had the largest effects (PVE  = 20.6% and 13.8%, respectively). The allele from the cultivated parent at the QTL on LG9 had the effect of decreasing STL, whereas the alleles from the cultivated parent at the other QTLs had the effect of increasing stem length.

Determinacy (STDET): Stem determinacy was characterized as a qualitative trait. The cultivated parent has a determinate growth habit and the wild parent has an indeterminate growth habit. The F_1_ plant showed an indeterminate growth habit, indicating that indeterminate growth habit from the wild parent was dominant. The segregation ratio for this trait fitted the expected ratio (1∶1). The gene controlling determinate growth habit from the cultivated parent is tentatively named *stdet5.9.1.* This gene was mapped between markers GATS11 and CEDG172 on LG9.

Stem thickness (STT): Only one QTL was detected on LG2 for stem thickness. At this QTL, the allele from the cultivated parent increased stem thickness. This QTL explained 10.2% of the phenotypic variation.

Branch number (BRN) and first branch position (BRP): The cultivated parent produced fewer branches on the main stem and initiated first branching at a higher internode than the wild parent (Table 4). Three QTLs for branch number (BRN) were identified on LG2, LG4 and LG6, and the QTL on LG2 had the largest effect (PVE  = 22.3%). At all QTLs, alleles from the cultivated parent had a negative effect on BRN. These QTLs were located close to those for flowering time (FLD) and days to pod maturity (PDDM). For first branch position (BRP), two QTLs with small effects were found on LG1 (11.5%) and LG5 (6.2%). At the QTL on LG5, the allele from the cultivated parent had a positive effect on BRP (*i.e.,* the first branch occurred at a higher node) whereas at the QTL on LG1, alleles from the cultivated parent had a negative effect on BRP.

Flowering time (FLD): The number of days to first flowering (FLD) was less in the cultivated parent than in the wild parent. Four QTLs for FLD were found on LG2, LG4, LG6 and LG11, and the QTL on LG2 had the largest effect (PVE  = 32.9%). The alleles from the cultivated parent had a positive substitution effect at three of the four QTLs (LG2, LG4 and LG6), but the allele from the cultivated parent had a negative substitution effect at the QTL on LG11.

Days to pod maturity (PDDM): The value for days to first pod maturity from first flowering (PDDM) was smaller in the cultivated parent (*i.e.*, time was shorter) than in the wild parent. Six QTLs for PDDM were found on LG2, LG4, LG6, LG7, LG9 and LG11. The QTLs with the largest effects were on LG2 and LG4 and explained 20.3% and 19.9% of the phenotypic variation, respectively. As expected, the alleles from the cultivated parent had a positive substitution effect at four of the QTLs (LG2, LG4, LG6 and LG9), but had a negative substitution effect at the other two (LG7 and LG11). The QTLs on LG2, LG4, LG6 and LG11 were located close to the four QTLs for FLD.

Seed number per pod (SDNPPD): The cultivated parent produced more seeds per pod than the wild parent (Table 4). Two QTLs with small effects (7.0% and 9.1%) for seed number per pod (SDNPPD) were identified on LG1 and LG9, respectively. At both QTLs, alleles from the cultivated parent had a positive effect on SDNPPD.

Pod total number (PDTN): The cultivated parent produced fewer total pods than the wild parent (Table 4). Four QTLs with small to intermediate effects (PVE  = 5.8%–12.0%) for pod total number (PDTN) were identified on LG2, LG4 (two QTLs) and LG7. At all QTLs, alleles from the cultivated parent had a negative effect on PDTN.

Seed pigmentation (SDC, SDCBM, and SDHC): Seed coat color (SDC), black mottle on seed coat (SDCBM), and hilum color (SDHC) were categorized as qualitative traits. The seed coat color of the cultivated parent is green whereas the wild parent is brown. The segregation ratio for this trait fitted the expected ratio (1∶1). The gene controlling green SDC from the cultivated parent is tentatively named *sdc5.5.1*. This gene was mapped between markers GMES4255 and CEDG020 on LG5 (Figure 2).

Black mottle on seed coat (SDCBM) is present in the wild parent and absent in the cultivated parent. The segregation ratio for this trait was distorted from the expected 1∶1 ratio (χ^2^ = 7.056; *P*<0.05). The gene controlling black mottle from the wild parent is tentatively named *sdcbm5.4.1*. This gene was mapped between markers GMES1820 and GMES0162a on LG4 (Figure 2).

The hilum color (SDHC) of the cultivated parent is white whereas the wild parent is pale red. The segregation ratio for this trait fitted the expected ratio (1∶1). The gene controlling white hilum from the cultivated parent is tentatively named *sdhc5.5.1*. This gene was closely linked to the gene controlling green SDC (*sdc5.5.1*) and was mapped between markers GMES4255 and CEDG020 on LG5 (Figure 2).

### Distribution of Bomestication-Related QTLs by Linkage Group

#### Linkage group 1

The QTLs for various traits were found in several regions of this chromosome (Figure 2). QTLs for traits related to loss of pod dehiscence and increased pod size were found in a narrow region of 8.0 cM between markers CEDCAA001a and cp04220. Also, a QTL for the loss of seed dormancy was found positioned about 15 cM from this region. Linkage group 1 appears to be important for the loss the pod dehiscence and seed dormancy. QTLs for increased seed size were found in two regions. QTLs for internode length (ST5I and ST6I) and stem length were found at the upper end of this linkage group.

#### Linkage group 2

Many QTLs were found in the region of about 20 cM between markers CEDG096a and CEDG026a (Figure 2). This region contained QTLs for various traits related to seed and stem size, flowering time, pod maturity, seed coat permeability, branch number, internode (ST9I and ST10I) and stem length. At a position about 10 cM from this region, the QTL for pod length was found.

#### Linkage group 4

Most of QTLs on this linkage group were distributed in a narrow region of about 10 cM between markers GMES0216a and MBSSE015a (Figure 2). In this region, there were QTLs for flowering time (FLD), pod maturity (PDDM), pod total number (PDTN) and branch number (BRN). The QTLs for FLD and PDDM each explained about 20% of the phenotypic variation for that trait, and the alleles from the cultivated parent shortened the days to flowering and pod maturity. The QTL for PDTN explained 12.0% of the phenotypic variation for this trait, and the allele from the cultivated parent decreased the total pod number.

#### Linkage group 7

Most of the QTLs on LG7 were concentrated in two regions (Figure 2). The first was a narrow region (7.2 cM) between markers GMES1424 and cp07863. The QTLs for seed and pod size were found in this region, and the alleles from the cultivated parent had the effect of increasing size. In particular, the QTLs related to pod size explained 20.5% (PDL) and 28.5% (PDW) of the phenotypic variation for these traits. The second region spanned 6.1 cM between markers MBSSR008 and CEDG111 and contained several QTLs related to pod dehiscence (PDR1W, PDR2W and PDR4W) and a QTL for pod number (PDTN). The alleles at these QTLs from the cultivated parent decreased pod dehiscence and pod number, respectively.

#### Linkage group 8

Several QTLs for seed and pod size were found in a narrow region of 4.3 cM between markers VM37 and CEDG059 (Figure 2). Most of these QTLs explained 6%–22% of the phenotypic variation for the corresponding trait, and the alleles from the cultivated parent increased size.

#### Linkage group 9

All of the QTLs and genes found on LG9 were distributed in a narrow region of 5.4 cM between markers GMES1216c and CEDG172 (Figure 2). The gene controlling indeterminate growth habit was mapped to this region, and the QTLs for 9^th^ and 10^th^ internode length and stem length were mapped nearby. Each of these QTLs explained over 20% of the phenotypic variation for the corresponding trait, and alleles from the cultivated parent decreased length.

#### Linkage group 10

The QTLs for each internode length and stem length found on this LG were distributed in a narrow region of 12.6 cM between markers CEDG116 and cp05914 (Figure 2). The QTLs for 5^th^ to 8^th^ internode length had large effects (PVE>20%). Alleles from the cultivated parent increased length.

Although it is unlikely that all 105 QTLs and genes identified for domestication-related traits have independent gene action on each trait, the observed number of QTLs and genes was compared with the expected number of QTLs and genes calculated on the basis of each linkage-group length (Table 5). The number of QTLs on LG7 was significantly higher than expected, but the total χ^2^ value across all 11 linkage groups was not significant at *P*  = 0.05. This suggests that the QTLs and genes for domestication-related traits are randomly distributed across the mungbean genome. To test whether the QTL distribution within each chromosome was random, the number of QTLs and genes within each 10-cM interval was counted and compared to the expected value by χ^2^ tests (Table 6). These χ^2^ tests indicated a non-random distribution of QTLs within each linkage group except for LG1, LG3, LG4 and LG5.

**Table 5:** Observed and expected number of QTLs on each linkage group and test of random distribution.

	Length	No. of QTL and gene			
LG	(cM)	Detected	Expected	X^2^	
1	91.0	16	13.1	0.63	
2	92.5	15	13.3	0.20	
3	74.1	9	10.7	0.27	
4	87.7	7	12.7	2.53	
5	57.2	3	8.3	3.34	
6	55.4	9	8.0	0.13	
7	48.2	13	7.0	5.25	*
8	69.1	8	10.1	0.39	
9	56.9	7	8.2	0.18	
10	50.4	11	7.3	1.91	
11	45.1	7	6.5	0.00	
Total	727.6	105	105.0	14.87	

* indicates significance at 5%.

**Table 6:** The number of QTLs and genes in each 10 cM interval on the mungbean map.

	No. of QTL and gene				
LG	Detected	Average^a^	Range^b^	X^2*c*^	
1	17	1.78	0–5	4.56	
2	15	2.00	0–8	145.55	***
3	9	1.13	0–3	5.38	
4	7	0.78	0–3	2.85	
5	3	0.50	0–2	1.06	
6	9	1.50	0–4	15.27	**
7	13	2.60	0–7	24.85	***
8	8	1.14	0–5	28.12	***
9	7	1.17	0–6	924.29	***
10	11	2.20	0–9	554.54	***
11	7	1.20	0–4	21.21	***

a The average QTL density on each linkage group.

b The range of the number of QTL and gene per 10 cM interval.

c Departure from random distribution of QTL and gene in each.

10 cM interval was tested under the null hypothesis in a Poisson.

goodness of fit.

** and *** indicate significance at 1% and 0.1% level,

respectively.

## Discussion

### General Features of the Mungbean Genetic Linkage Map

The present mungbean map is the first in which the number of linkage groups coincided with the haploid chromosome number of mungbean (n  = 11). A total of 430 SSR markers loci from azuki bean, mungbean, cowpea and common bean and EST-SSR markers from soybean are included on this map. Most of the duplicated loci were detected from soybean EST-SSR markers. This may be because of lower annealing temperature in PCR reaction for these markers. The marker density in this map is the highest among the mungbean maps that have been reported to date, and there are no gaps of greater than 20 cM. Therefore, this mungbean map is well suited for the identification of QTLs for domestication-related traits and other agronomically important characters. It will also be useful for the analysis of genome synteny among species within the genus *Vigna*.

In this mungbean map, an SSR marker designed from the bruchid resistance gene *Vigna radiata defensin 1* (*VrD1*) [30] was mapped to the upper region of LG1. On the other hand, Young et al. [16] and Kaga and Ishimoto [17] mapped bruchid resistance gene *Br1* to LG8 of the map by Menancio-Hautea et al. [10], which corresponds to LG2 of this mungbean map. Therefore, the mapping data show that the gene encoding resistance protein VrD1 differs from the bruchid resistance gene *Br1* reported by Kitamura et al. [46].

### Segregation Distortion

Segregation distortion (*P*<0.05) was observed for 61 markers (14.4%). Most of the markers on LG4 showed a high level of segregation distortion (*P*<0.001) toward a higher than expected frequency of the wild-parent allele. LG4 harbors important domestication QTLs related to seed dormancy, seed productivity and daylength sensitivity. In *V. radiata,* plants that were classified as an intermediate weedy form between the cultivated and wild mungbean are frequently found growing naturally in India, Indonesia, Nepal, China, and Japan [28]. Segregation distortion toward wild alleles, as observed for LG4 in the present study, may promote increased survival of plants derived from natural hybridization and allow them to develop natural weedy populations.

### Genomic Regions and Distributions of Qtls Involved in Mungbean Domestication

As shown in many legume crops such as common bean [47], soybean [48], azuki bean [23], [24], pea [49] and rice bean [25] and graminaceous crops such as maize [50], rice [51] and pearl millet [52], domestication-related traits are generally controlled by a few major genes plus some minor genes, and these genes are distributed within narrow regions on a small number of linkage groups. As in other crops, domestication-related traits in mungbean were found to be controlled by a few major genes plus some minor genes (Table S3), and these were distributed within narrow regions on linkage groups (Figure 2 and Table 6). However, many more linkage groups have been involved in the domestication of mungbean than in other crops: 20 major QTLs (PVE≥20%) for domestication-related traits were distributed on seven of the 11 linkage groups (Figure 2 and Table S3). These numbers of major QTLs and linkage groups are the largest reported for species in the subgenus *Ceratotropis* (azuki bean: [23], [24]; rice bean: [25]). This indicates that mutations causing major changes in morphological and physiological traits desirable for human use have occurred on more linkage groups in mungbean than in other related species. In addition, a total of 53 QTLs with small effects (PVE<10%) were also found on each linkage group (Table S3). The accumulation of many mutations with large and small effects may have resulted in the differentiation between wild and cultivated mungbean, which is considered the most highly domesticated crop of the Asian *Vigna* cultigens.

### QTLs for 100-Seed Weight

In this study, seven QTLs for 100-seed weight (SD100WT) were found on six linkage groups (Figure 2 and Table S3). Fatokun et al. [8] identified four QTL regions for seed weight, and Humphry *et al.* [14] found a total of 11 QTLs for seed weight. Unfortunately, a comparison of QTL regions between this study and that of Humphry et al. [14] was not possible because the kinds of markers used to construct the linkage map were different. On the other hand, QTLs on LG1 (*Sd100wt5.1.2*) and LG8 (*Sd100wt5.8.1*) in this study appeared to correspond to those on LG vi and LG ii, respectively, reported by Fatokun et al. [8]. The remaining five QTLs did not appear to correspond to any reported by Fatokun et al. [8].

Two QTLs for SD100WT on LG1 (*Sd100wt5.1.2*) and LG2 (*Sd100wt5.2.1*) appeared to correspond to QTLs identified in azuki bean [23], [24] and rice bean [25]. Furthermore, the QTLs for SD100WT on LG3 (*Sd100wt5.3.1*) and LG7 (*Sd100wt5.7.1*) appeared to correspond to a QTL in rice bean [25], and the QTL for SD100WT on LG11 (*Sd100wt5.11.1*) appeared to correspond to a QTL in azuki bean [24]. In particular, the QTLs on LG2 had the largest effect on SD100WT in azuki bean and the second largest effect in mungbean and rice bean; hence, this locus appears to have contributed substantially to the seed size increase that has occurred during *Vigna* domestication. These results suggest that mutations for seed size occurring at the same locus have been selected by farmers during the independent domestication processes of these legumes. Interestingly, the QTL with the largest effect (22.2%, Table S3), *Sd100wt5.8.1* on LG8, was specific to mungbean, and thus this QTL might be useful for breeding of varieties with larger seeds in other *Vigna* crops such as black gram, rice bean and azuki bean.

### QTLs for Seed Dormancy

In Asian *Vigna* species, water enters the seed through the lens (strophiole) near the hilum [53], [54]. To determine the genetic factors related to physical dormancy in mungbean, the water absorption by seeds (SDWA) was evaluated and four QTLs were identified on four linkage groups (Figure 2 and Table S3). Out of these QTLs, *sdwa5.1.1* on LG1 had the largest effect (33.7%, Table S3). Therefore, the structure of the lens (strophiole) may be controlled by one or more genes in this QTL region. Two QTLs for SDWA were identified in azuki bean [23], [24] and five in rice bean [25]. Interestingly, no common QTLs for this trait were found among these three species.

High-quality mungbean seeds are difficult to produce in humid tropical regions because of susceptibility to field-weathering damage. Hard-seededness can reduce the level of damage [14], but attempts to breed large- and hard-seeded mungbean varieties have not been successful. Humphry et al. [14] showed that QTLs for hard-seededness and seed weight were co-localized on two linkage groups and that alleles associated with hard-seededness and low seed weight were usually inherited together. Similarly, a positive and significant correlation (*r* = 0.498; *P*<0.001) between 100-seed weight (SD100WT) and water absorption by seeds (SDWA) was also observed in the present study (Table S2), and tight linkages between QTLs for SD100WT and SDWA were found on LG1, LG2 and LG3 (Figure 2 and Table S3). Therefore, it is necessary to elucidate whether the effects of these QTLs are caused by pleiotropic effects of the same gene or by tightly linked but distinct genes. If the effects are proved to be caused by tight linkage rather than pleiotropy, it may be possible to separate the traits using marker selection.

### QTLs for Pod Dehiscence

The loss of seeds by pod dehiscence is one of the major reasons for low yield in mungbean; thus, reducing the frequency of pod dehiscence is an important objective in mungbean breeding [55].

In this study, two QTL regions associated with the loss of pod dehiscence were identified on LG1 and LG7. For other *Ceratotropis* species, only one QTL (on LG7) was found in azuki bean [23], [24] and in rice bean [25]. The QTL region on LG7 detected in this study appeared to be the same as those in the other two species, but the QTL region on LG1 was specific to mungbean. This newly identified mutation on LG1 might play an important role in improving pod dehiscence of other *Vigna* crops.

One minor QTL region with an allele from the cultivated parent that accelerated pod dehiscence was found on LG6 by evaluating the frequency of pod shattering (PDR1W, PDR2W, PDR4W: PVE  = 4.5%–6.1%). The wild allele at this locus may be useful for improving pod dehiscence of the cultivated mungbean.

### QTLs for Traits Related to Plant Type

Major QTLs for middle internode length (ST5I–ST8I) were found on LG10, and the alleles from the cultivated parent increased these internode lengths (Figure 2 and Table S3). On the other hand, the main QTLs for upper internode length (ST9I and ST10I) were found on LG9, and the alleles from the wild parent increased the length of these internodes (Figure 2 and Table S3). Thus it appears that the genetic control of internode length shifts from a gene on LG10 to a gene on LG9 at higher positions on the plant. A similar phenomenon was reported in azuki bean, and the QTLs for upper internode length on LG9 appeared to be common to those in azuki bean [23], [24].

The gene controlling indeterminate growth habit from the cultivated parent (*stdet5.9.1*) was mapped between markers GATS11 and CEDG172 on LG9, and its gene position coincided with QTL positions for ST9I, ST10I and STL (Figure2). This gene position also coincides with a QTL position reported for the loss of twining habit in azuki bean [23], [24]. Therefore, the genome region controlling growth habit may be conserved between mungbean and azuki bean. On the other hand, three SSR markers that were associated with determinacy in soybean were mapped on LG4 (LFsoy3), LG5 (LFsoy5) and LG6 (LFsoy9), even though these regions were not associated with determinacy in this study. This suggests that different mutations (genes) control determinacy in mungbean and soybean.

Three QTLs for branch number (BRN) were found on LG2, LG4 and LG6, and the alleles from the cultivated parent decreased branch number (Figure 2 and Table S3). No relationship between branch number and stem length (including individual internode lengths) was observed. On the other hand, QTLs for days to flowering (FLD) were found near these QTLs (Figure 2 and Table S3). A significant positive correlation between branch number and days to flowering (*r* = 0.490, *P*<0.001) was observed (Table S2).

### QTLs for Phenology-Related Traits

QTLs for decreased number of days to flowering (FLD) were found on LG2, LG4 and LG6. The QTLs *Fld5.2.1* on LG2 and *Fld5.4.1* on LG4 explained 32.9% and 24.0% of the phenotypic variation, respectively (Figure 2 and Table S3). The QTLs corresponding to those on LG2 and LG4 were also found in azuki bean [23], [24]. This suggests that mutations for the loss of sensitivity to short daylength occurred in the same genome regions as those in azuki bean during the process of domestication. Mungbean cultivation may have expanded to higher-latitude regions such as China and Japan following the loss of the sensitivity to short daylength.

As observed in azuki bean [24] and rice bean [25], it is reasonable to expect that the number of days from flowering to pod maturity would be greater in a cultivated parent with large seeds than in a wild parent with small seeds. However, in mungbean the number of days to pod maturity in the cultivated parent was less than in the wild parent (Table 4), and four QTLs with alleles from the cultivated parent that accelerated pod maturity (PDDM) were found on LG2, LG4, LG6 and LG9 (Figure 2 and Table S3). In addition, a significant negative correlation (*r* = −0.17; *P*<0.01) between PDDM and 100-seed weight (SD100WT) was observed (Table S2), and the major QTL for PDDM on LG2 was closely linked to the QTL for SD100WT (Figure 2 and Table S3). The QTLs for PDDM on LG2, LG4 and LG6 were closely linked to those for FLD, and the alleles from the cultivated parent accelerated flowering. Therefore, in addition to the shortening of days to flowering, the shortening of days to pod maturity may have contributed to the establishment of mungbean as a short-season crop.

Two QTLs on LG7 (*Pddm5.7.1*) and LG11 (*Pddm5.11.1*) were also related to the duration necessary for pod maturity. Although the variation explained by these QTLs is not high, the wild alleles at these loci were associated with a shorter duration needed for pod maturity, hence, the use of these alleles may contribute to the breeding of more rapidly maturing mungbean cultivars.

### Genes for Organ Color

The presence or absence of black mottle on the seed coat (SDCBM), seed coat color (SDC), and hilum color (SDHC) were controlled by single genes. The gene (*sdcbm5.4.1*) controlling SDCBM from the wild mungbean was mapped between markers GMES1820 and GMES0162a on LG4. It was previously reported that the presence of SDCBM in wild mungbean was controlled by a single dominant gene [19], [56]. This gene was mapped on linkage group 2 of the map by Lambrides et al. [11], which corresponds to LG4 of the map in this study, and the gene position coincided between both maps. Moreover, in several azuki bean maps [23], [24], [57], the gene controlling SDCBM was located on linkage group 4, and these gene positions coincided with that of mungbean. Therefore, the gene controlling SDCBM may be highly conserved among the Asian *Vigna* species.

The gene (*sdhc5.5.1*) controlling white hilum color (SDHC) from cultivated mungbean was mapped between markers GMES4255 and CEDG020 on LG5. It was previously reported that the white hilum color from cultivated mungbean was recessive to pale-red color from wild mungbean, and that this trait was controlled by a single gene [56]. In rice bean [25], the gene controlling white hilum color from cultivated rice bean was mapped on LG5 at a position coinciding with that of *sdhc5.5.1* in the present study.

### Plans for the Future

Although EST-SSR markers from soybean were mapped on the mungbean map in this study, detailed comparison between the genomes of those two species was not possible because the number of common markers between the mungbean and soybean maps [42], [58] was very small. We are continuing the mapping of EST-SSR markers from soybean on maps of azuki bean, rice bean and black gram. We will construct a consensus map based on the four Asian *Vigna* legumes maps to elucidate the genome synteny between the Asian *Vigna* legumes and soybean.

### Conclusions

A genetic linkage map of mungbean has been constructed using 237 SSR markers from mungbean and closely related species and 193 EST-SSR markers from soybean. The present mungbean map is the first map in which the number of linkage groups coincided with the haploid chromosome number. In total, 105 QTLs and genes for 38 domestication-related traits were identified, and QTLs with large effects (PVE≥20%) were distributed on seven out of 11 linkage groups. In addition, cultivated mungbean has accumulated a large number of QTLs with small effects (PVE<10%). As a result, the accumulation of many mutations with both large and small effects may have led to the differentiation between wild and cultivated mungbean, and it is thought that the degree of domestication in mungbean is the highest among the Asian domesticated *Vigna* species. For seed size, seed dormancy, pod dehiscence, and pod maturity, QTLs that were not found in other Asian *Vigna* species were identified in mungbean, and these QTLs may be useful new gene resources for other Asian *Vigna* species. These results provide a foundation genetic map that will be useful for the improvement of mungbean and related legumes.

## Supporting Information

Figure S1 **Frequency distribution for traits examined in the BC_1_F_1_ (or BC_1_F_1∶2_) populations.** In this Figure, frequency distribution for 20 traits (SD100WT, SDL, SDT, SDW, SDWA, SDNPPD, PDR1W, PDR2W, PDR4W, PDT, PDL, PDW, PDTN, LFPL, LFPW, LFML, LFMW, STT, HECL and ST1I: Trait abbreviations are shown in Table 1.) are shown. The letters “C” and “W” indicate the cultivated and wild mungbean, respectively. The bars and horizontal lines under the letters “C” and “W” indicate the position of mean and standard error, respectively.
(TIF)

Figure S2 **Frequency distribution for traits examined in the BC_1_F_1_ (or BC_1_F_1∶2_) populations.** In this Figure, frequency distribution for 14 traits (ST2I – ST10I, STL, BRN, BRP, FLD and PDDM: Trait abbreviations are shown in Table 1.) are shown. The letters “C” and “W” indicate the cultivated and wild mungbean, respectively. The bars and horizontal lines under the letters “C” and “W” indicate the position of mean and standard error, respectively.
(TIF)

Table S1 **The primer information on SSR and EST-SSR markers mapped in this study.**
(XLS)

Table S2 **Correlation coefficients among each trait in the BC_1_F_1_ (or BC_1_F_1∶2_) populations derived from a cross between cultivated and wild mungbean.**
(XLS)

Table S3 **QTLs detected in BC_1_F_1_ (or BC_1_F_1∶2_) populations derived from a cross between cultivated and wild mungbean.** Trait abbreviations are shown in Table 1. PVE: phenotypic variation explained.
(XLS)

Dataset S1 **The genotype data for 430 SSR and EST-SSR markers in each BC_1_F_1_ plant.**
(XLS)

Dataset S2 **The phenotype data for 38 domestication related traits in each BC_1_F_1_ plant or BC_1_F_1∶2_ line.**
(XLS)
