# Supplementary figures and images for: Construction of a Genetic Linkage Map and Genetic Analysis of Domestication Related Traits in Mungbean (Vigna radiata)
Source: PLoS One. 2012 Aug 2;7(8):e41304. doi: 10.1371/journal.pone.0041304 (PMC3410902; doi:10.1371/journal.pone.0041304)

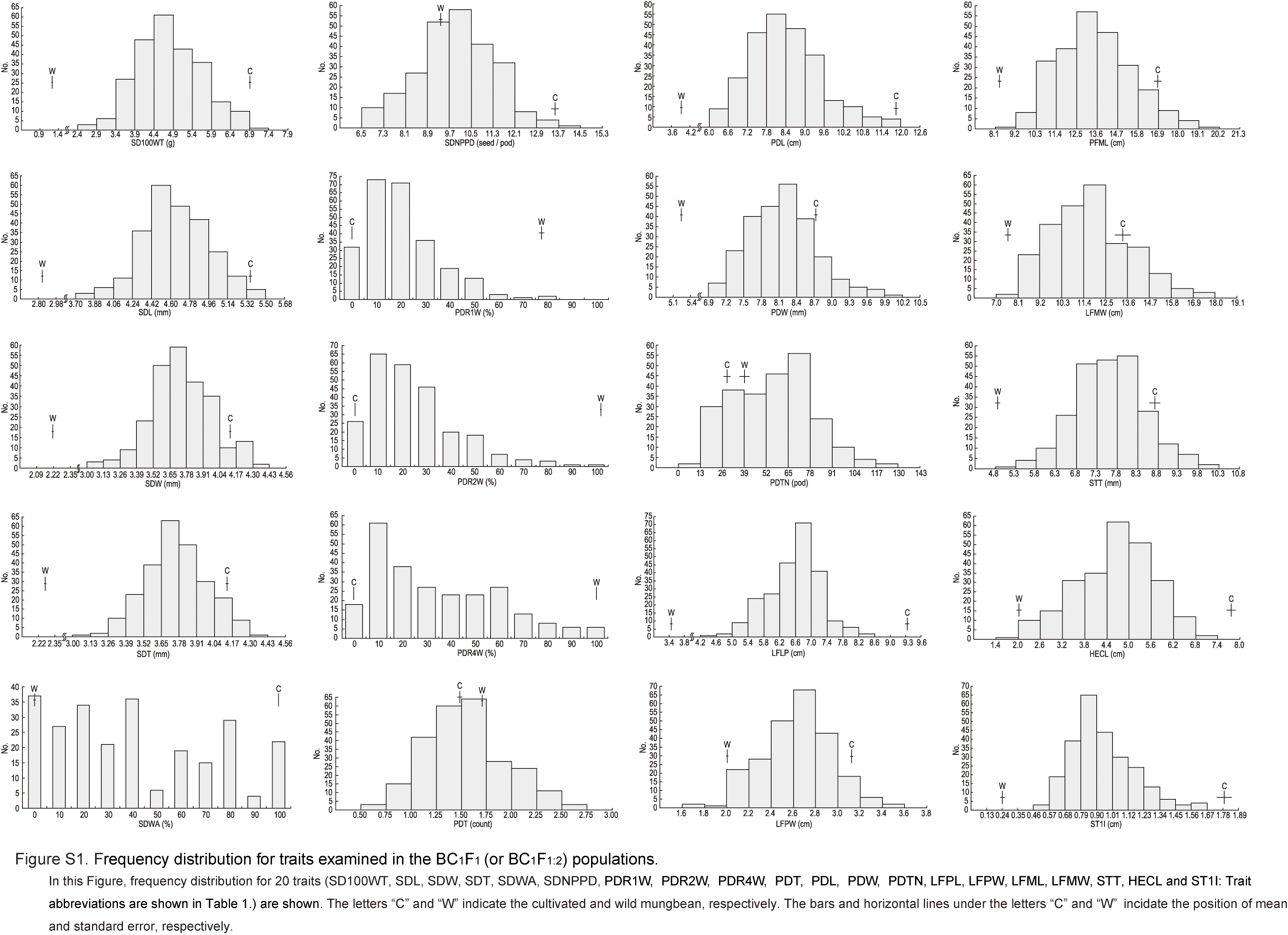

Supplement: Figure S1 — Frequency distribution for traits examined in the BC1F1 (or BC1F1∶2) populations. In this Figure, frequency distribution for 20 traits (SD100WT, SDL, SDT, SDW, SDWA, SDNPPD, PDR1W, PDR2W, PDR4W, PDT, PDL, PDW, PDTN, LFPL, LFPW, LFML, LFMW, STT, HECL and ST1I: Trait abbreviations are shown in Table 1.) are shown. The letters “C” and “W” indicate the cultivated and wild mungbean, respectively. The bars and horizontal lines under the letters “C” and “W” indicate the position of mean and standard error, respectively. (TIF) [file pone.0041304.s001.tif]

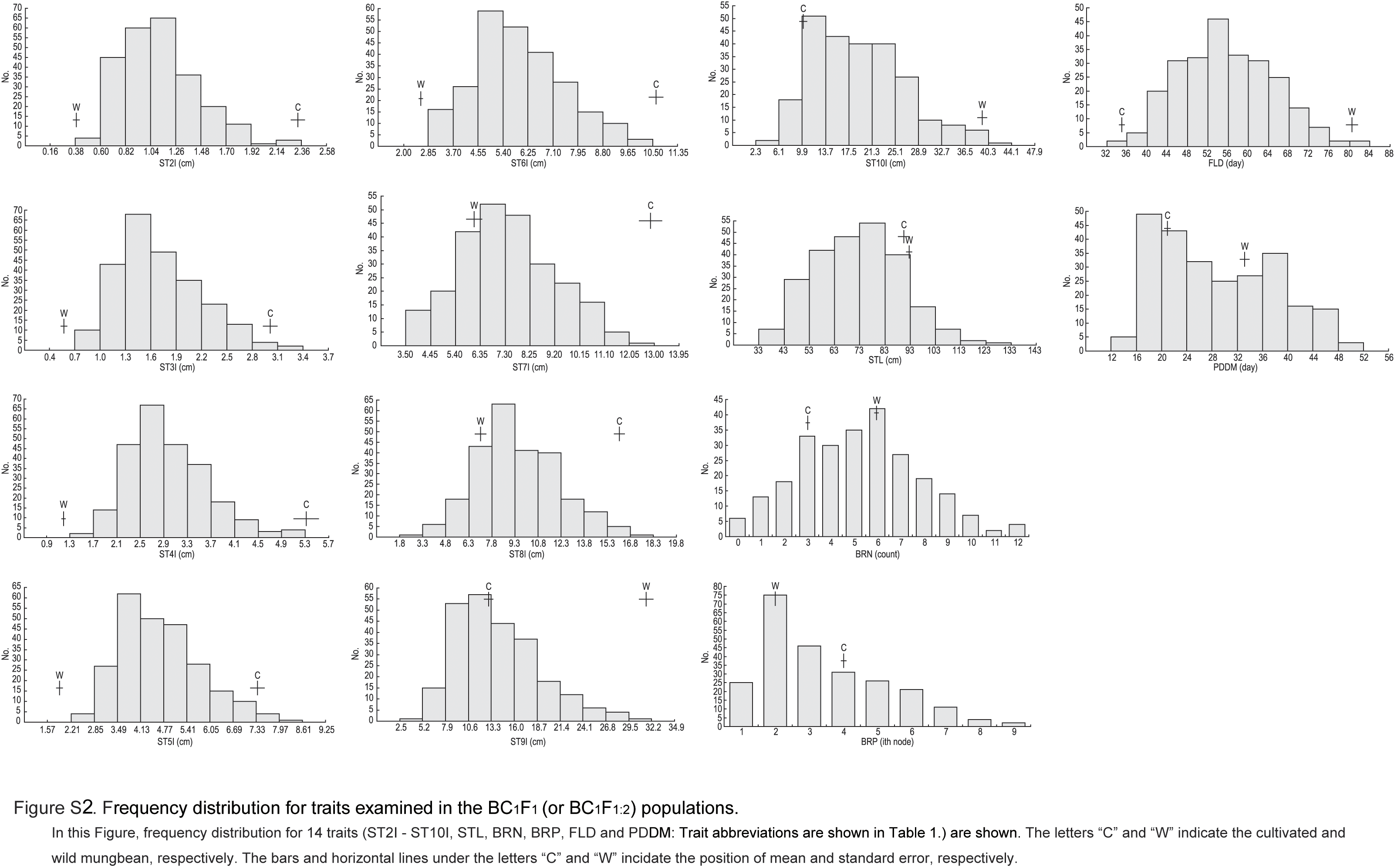

Supplement: Figure S2 — Frequency distribution for traits examined in the BC1F1 (or BC1F1∶2) populations. In this Figure, frequency distribution for 14 traits (ST2I – ST10I, STL, BRN, BRP, FLD and PDDM: Trait abbreviations are shown in Table 1.) are shown. The letters “C” and “W” indicate the cultivated and wild mungbean, respectively. The bars and horizontal lines under the letters “C” and “W” indicate the position of mean and standard error, respectively. (TIF) [file pone.0041304.s002.tif]
